# Characterization of Endogenous Levels of Brassinosteroids and Related Genes in Grapevines

**DOI:** 10.3390/ijms23031827

**Published:** 2022-02-05

**Authors:** Francisca Parada, Jana Oklestkova, Patricio Arce-Johnson

**Affiliations:** 1 Laboratorio de Biología Molecular y Biotecnología Vegetal, Departamento de Genética Molecular y Microbiología, Facultad de Ciencias Biológicas, Pontificia Universidad Católica de Chile, Santiago 8320000, Chile; 2 Centro de Biología Molecular Vegetal, Departamento de Biología, Facultad de Ciencias, Universidad de Chile, Santiago 7750000, Chile; 3 Laboratory of Growth Regulators, Institute of Experimental Botany of the Czech Academy of Sciences & Faculty of Science, Palacký University, 77200 Olomouc, Czech Republic; jana.oklestkova@upol.cz

**Keywords:** brassinosteroids, grapevine, development, UHPLC-MS/MS

## Abstract

Agronomic breeding practices for grapevines (*Vitis vinifera* L.) include the application of growth regulators in the field. Brassinosteroids (BRs) are a family of sterol-derived plant hormones that regulate several physiological processes and responses to biotic and abiotic stress. In grapevine berries, the production of biologically active BRs, castasterone and 6-deoxocastasterone, has been reported. In this work, key BR genes were identified, and their expression profiles were determined in grapevine. Bioinformatic homology analyses of the Arabidopsis genome found 14 genes associated with biosynthetic, perception and signaling pathways, suggesting a partial conservation of these pathways between the two species. The tissue- and development-specific expression profiles of these genes were determined by qRT-PCR in nine different grapevine tissues. Using UHPLC-MS/MS, 10 different BR compounds were pinpointed and quantified in 20 different tissues, each presenting specific accumulation patterns. Although, in general, the expression profile of the biosynthesis pathway genes of BRs did not directly correlate with the accumulation of metabolites, this could reflect the complexity of the BR biosynthesis pathway and its regulation. The development of this work thus generates a contribution to our knowledge about the presence, and diversity of BRs in grapevines.

## 1. Introduction

Modern grapevine (*Vitis vinifera* L.) cultivation (viticulture) has focused on the management and improvement of this crop with cultural strategies such as the use of rootstocks, optimization of pruning techniques, and the applications of fertilizers and phytohormones [1,2]. The addition of phytohormones is frequently employed to boost and/or synchronize development, and to increase tolerance to environmental challenges, often by mimicking the roles of endogenous hormones present in the plant itself [3,4,5]. Of these, brassinosteroids (BRs) are a family of polyhydroxylated steroidal plant hormones, discovered in pea seedlings (*Pisum sativum* L.). BR concentrations in plants are very low, typically representing just 10^−12^–10^−9^ g/g of the fresh weight in plants, generating technical difficulties for their isolation [6]. Fortunately, newer and more sensitive methods have been generated, such as immunoaffinity purification and UHPLC-ESI-MS/MS quantification, significantly facilitating their detection, identification and quantification [7,8].

BRs play a key role during biotic and abiotic stress responses. For example, brassinolide improves resistance to viral, bacterial, and fungal pathogens in rice, tobacco, and strawberry through the modulation of innate immune system responses [9,10]. For this reason, the use of BRs has been proposed as an agrochemical alternative for improving crop protection [10]. Furthermore, it has been reported that BRs enhance tolerance to several types of abiotic stresses such as drought, cold, salinity, and heavy metal stress, among others [11,12]. It has been shown that BRs improve CO_2_ assimilation, increase the production of antioxidant compounds, participate in reactive oxygen species (ROS) scavenging, and interact with the signaling pathway of the stress-related hormone abscisic acid (ABA) [11,13]. On the other hand, BRs participate during development processes such as skotomorphogenesis, vascular differentiation, stomatal development, senescence control, and flowering, among others [14,15]. Their most significant biological effect is the stimulation of growth through the regulation of cell division, cell expansion, and microtubule orientation [16,17,18,19].

The biosynthesis of BRs requires multiple oxidation steps, which are usually catalyzed by cytochrome P450 enzymes (P450s or CYPs) from 24-methylene cholesterol or another precursor. Mutants deficient in P450s and 5-reductase involved in the synthesis of BRs have been identified. Mutants deficient in BRs, in light conditions, have a dwarf phenotype with dark and folded green leaves, prolonged life cycle, reduced fertility, and altered vascular development, while in the darkness they show shorter hypocotyls and open cotyledons [20]. These phenotypes can only be reverted by the exogenous application of BRs [20].

BR bioactivity depends on the structure of the A or B rings and side chain [21,22,23,24]. Among those with the same ring structure, BR biological activity has been ranked as follows in decreasing order: (1) 22α and 23α-dihydroxybrassinosteroids are as active as 28-homobrassinosteroids and possess the highest bioactivity, (2) 24-epibrassinosteroids or 28-norbrassinosteroids, and (3) 26-norbrassinosteroids [24,25,26,27,28]. The presence and quantity of these BRs is species- and tissue-dependent [6]. In Arabidopsis, castasterone (CS) and brassinolide (BL) are produced, with BL being the most active [29]. In the case of tomato, CS is the most bioactive BR in vegetative tissue and 6-deoxoCS in fruits [29]. On the other hand, BL has not been detected in rice (*Oryza sativa*), and it uses CS as the active BR [30,31].

The best-known BR biosynthesis routes are the “Early C-22 oxidation”, “Late C-6 oxidation”, and “Early C-6 oxidation” pathways using campesterol as precursor. The early C-6 oxidation route generates BL as a terminal product, a compound that has the highest bioactivity among the BRs [32,33]. Additionally, another route allows the synthesis of C27 BRs using cholestanol as precursor and 28-norcastasterone as terminal product [33,34]. Alternatively, sitosterol is used to produce 28-homocastasterone (HCS) [35]. The production of 28-homobrassinolide from HCS has also been reported [36]. To date, many enzymatic steps and intermediates in these routes are unknown. Based on the studies conducted so far, in Arabidopsis, peas, and tomatoes, it has been determined that the late oxidation pathway of C-6 is predominant [37], which has also been suggested in grapevines [38].

Exogenous applications of 24-epibrassinolide (24-epiBL) have been used broadly in grapevine berries in different studies, where an increase in ripening parameters such as anthocyanin content, total soluble sugars and phenolic compounds has been found [39,40,41,42]. Moreover, an increase in trans-resveratrol, α-carotene, and ascorbic acid were reported, suggesting a role for BRs in the production of pigment and antioxidant compounds in grapes [43].

Besides the treatments with exogenous BRs, high endogenous concentrations of CS and its direct precursor, 6-deoxoCS, have been quantified during the veraison and ripening stages of grapevine, suggesting a role in maturation [38]. Regarding the biosynthesis pathway of BRs, *VviBR6OX1* encodes BRASSINOSTEROID-6-OXIDASE 1 (BR6OX1/CYP85A1) that catalyzes the conversion of 6-deoxoCS to CS and was functionally characterized by complementation of mutant lines of tomato [38].

Many of the studies performed to identify new genes of the BR biosynthesis pathway, perception and signaling in different plants, have been carried out by heterologous complementation of rice, tomato, and Arabidopsis mutants for these genes. Studies so far suggest that the molecular mechanism of these pathways is conserved between monocotyledonous and dicotyledonous plants [31,44,45,46,47,48,49]. Accordingly, preliminary bioinformatic analyses that we performed in the reference genome of *V. vinifera* L. uncovered several of the genes involved in these pathways with high identity to those described in Arabidopsis, suggesting that routes in grapevines could also be conserved. Moreover, the bioinformatic analysis of global expression data reported in the *V. vinifera* atlas [50] suggested that all these gene models are expressed in various tissues.

Although advances have been made in understanding the role of BRs in the maturation of the grapevine berry, information on this species is still scarce. The characterization of the biosynthesis, perception and signaling pathways, as well as the understanding of the changes in transcripts and metabolites regulated by these routes, are under-explored areas. In this work, a characterization of the level of metabolites of the BR family was carried out in different tissues and stages of grapevine development. In addition, the expression of the key genes involved in the biosynthesis, perception, and signaling pathways of BRs in grapevines was analyzed. To our knowledge, this is the broadest analysis of these phytohormones performed in grapevines to date.

## 2. Results

### 2.1. Quantification of the Levels of Brassinosteroids during the Development of Grapevines

**Figure 1:**
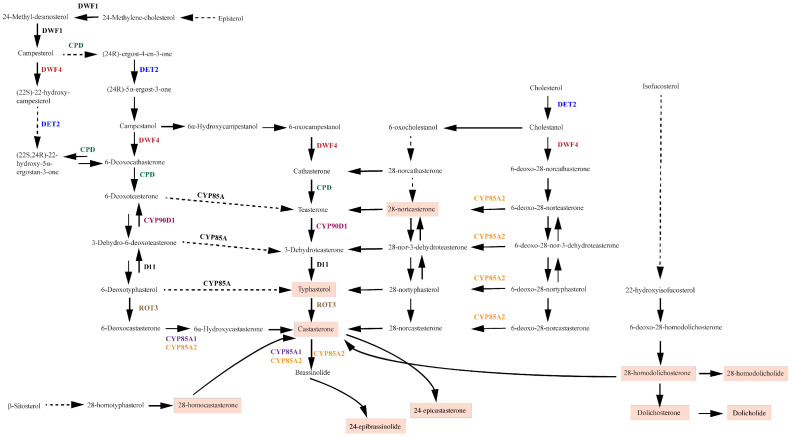
Brassinosteroid biosynthesis routes. The different routes of biosynthesis of brassinosteroids are depicted. Dashed lines indicate multiple enzymatic steps. Compounds detected in this work are highlighted in orange. Diagram based on [33,34,35,51,52,53].

In several grapevine tissues BRs were measured to establish accumulation patterns. The UHPLC-MS/MS technique has been previously described for the quantification of 15 different types of BRs [7]. Following this protocol, we were able to quantify ten types of BRs in grapevine samples (dolichosterone (DS), 28-norteasterone (28-norTE), homocastasterone (HCS), homodolichosterone (HDS), dolicholide (DL), homodolicholide (HDL), typhasterol (TY), 24-epicastasterone (24-epiCS), castasterone (CS), and 24-epibrassinolide (24-epiBL)). Other BR species were not detected in this plant material. The compounds found are highlighted in Figure 1.

**Figure 2:**
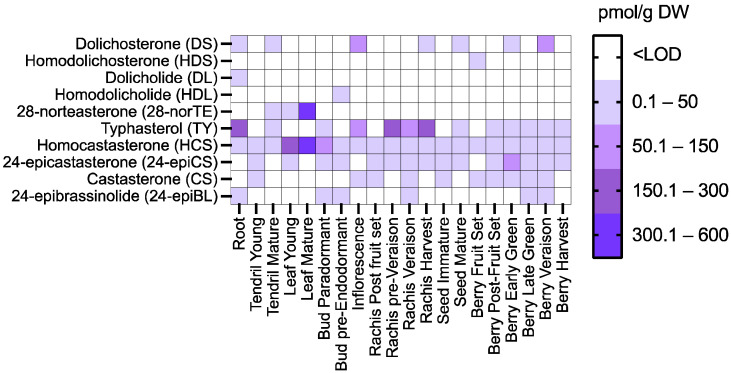
Quantification of brassinosteroids (pmol/g DW) in cv. Cabernet sauvignon grapevine tissues during development. Brassinosteroids were measured by UHPLC-MS/MS, and results were expressed as a heat map. Three biological samples and three technical replicates were measured, with each biological replicate corresponding to a pool of tissue collected from five different plants.

The majority of the detected BRs accumulate to concentrations lower than 50 pmol/g DW (Figure 2, Tables S1 and S2). The most widely distributed BR is HCS, a compound detected in all tissues and developmental stages analyzed. The highest concentrations of BRs are found in mature leaves with 535 pmol/g DW of 28-norTE, and 455.88 pmol/g DW of HCS (Figure 2, Tables S1 and S2). The metabolites of the CS group seem to be the most prevalent in grapevines since HCS, 24-epiCS and CS are differentially accumulated in all tissues studied (Figure 2, Tables S1 and S2). Grapevine roots, rachis at pre-veraison, and rachis at harvest harbor 215.72 pmol/g DW, 214.53 pmol/g DW, and 198.89 pmol/g DW of TY, respectively. It is interesting to highlight that BL, the terminal and most bioactive known BR, is not detected in any tissue, but its epimeric form, 24-epiBL, does accumulate in roots, paradormant and pre-endodormant buds, rachis at veraison, and berries in late green and veraison stage (Figure 2, Tables S1 and S2). During fruit set, berries mainly accumulate HDS at 49.87 pmol/g DW, in post-fruit set TY is the most prevalent BR (30.76 pmol/g DW), in the early green stage, the major metabolites present are 24-epiCS (56.69 pmol/g DW) and DS (39.11 pmol/g DW), during late green phase, the key BRs are TY (27 pmol/g DW) and 24-epiCS (15.87 pmol/g DW), in veraison DS (53.39 pmol/g DW) is the most abundant BR, whilst at harvest TY was found at 11.94 pmol/g DW. These results highlight that BR synthesis produces an array of some of the 62 known compounds belonging to this phytohormone family, and that reproductive and vegetative tissues harbor different BR profiles.

### 2.2. Gene Expression Analysis of the Brassinosteroid Pathways in Grapevines

**Figure 3:**
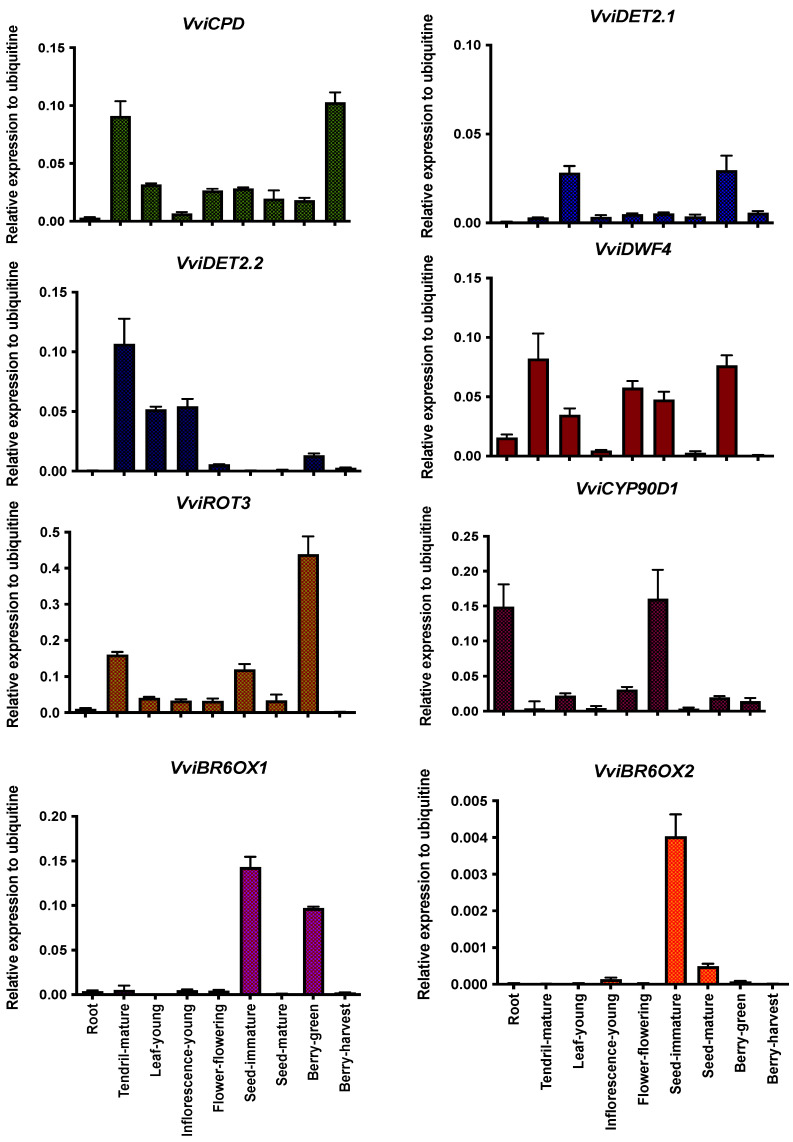
Expression levels of genes involved in brassinosteroid biosynthesis in cv. Cabernet sauvignon grapevines. Expression data was normalized against the reference gene, *VviUBI*. Bars are the mean ± standard deviation of three biological and three technical replicates. Each biological replicate corresponds to a pool of tissue collected from five different plants.

In order to further our understanding of this pathway, we then analyzed the expression patterns of key grapevine BR gene orthologues. To do so, seven genes of the BR biosynthetic pathways were found in the grapevine genome, all with a high percentage (>67%) of amino acid identity compared to those described in Arabidopsis. In the case of *VviDET2*, two gene models were identified and named *VviDET2.1* and *VviDET2.2*. The seven biosynthetic genes analyzed here have been described in different species and are known to be involved in several enzymatic steps for producing different BRs. These genes are highlighted with the same color code in both Figure 1 and Figure 3. It is essential to point out that each gene participates in several points of the biosynthetic routes, so the target genes that were analyzed in our research covered most of the previously described pathways. Additionally, a homologous gene to the D11 gene described in rice and maize [54,55] was not found in the grapevine genome analysis. All gene models are shown in Supplementary Table S3, as well as the properties of the primers designed for the expression analysis. The sequences of selected genes are shown in Supplementary Table S4.

The expression profiles for genes of the biosynthesis routes for BRs were analyzed in different tissues during grapevine development. CYP90A1 is involved in early stage C-3 oxidation of BR biosynthesis and is encoded by CPD [56]. Our analysis shows that *VviCPD* (VIT_213s0067g00660) is mainly expressed in mature tendrils, and in berries at the harvest stage (Figure 3). In the case of *VviDET2.1* and *VviDET2.2*, both encode putative steroid 5-reductase enzymes, acting at the second step of the BL biosynthesis path [57]. *VviDET2.1* (VIT_213s0067g01830) is mostly expressed in young leaves and green berries, while *VviDET2.2* (VIT_219s0014g00080) is preferentially expressed in mature tendrils, young inflorescences, young leaves, and green berries (Figure 3). *VviDWF4* (VIT_204s0023g01630) encodes a putative cytochrome CYP90B1 that catalyzes one of the most critical enzymatic steps, maintaining homeostasis of BRs [58,59], and is expressed in almost all tissues analyzed. Interestingly, while in immature seeds and green berries high levels of *VviDWF4* transcripts were found, they drop to nearly undetectable levels in the mature stages of these tissues (Figure 3). Moreover, *VviDWF4* is expressed at high levels in mature tendrils, flowers at the flowering stage, and young leaves. Genes *VviROT3* (VIT_204s0023g02650) and *VviCYP90D1* (VIT_209s0002g02080) encode for cytochrome P450 enzymes ROTUNDIFOLIA3/CYP90C1 and CYP90D1, respectively [60]. These enzymes may participate in intermediate oxidation steps in BR biosynthesis, where ROT3/CYP90C1 could catalyze the conversion of TY to CS and 6-DeoxoTY to 6-DeoxoCS [60]. On the other hand, CYP90D1 could catalyze the production of 6-Deoxo3DT and/or 3-Dehydroteasterone (3DT) to TE and 6-DeoxoTE, respectively [60]. *VviROT3* is mainly expressed in mature tendrils, immature seeds, and green berries while *VviCYP90D1* has higher expression levels in immature seeds and roots (Figure 3). The penultimate step for BL biosynthesis via both late and early oxidation routes involves the conversion of 6-DeoxoCS into CS. It is catalyzed by brassinosteroid-6-oxidase 1 (BR6OX1) [46]. In the case of grapevines, *VviBR6OX1* (VIT_214s0083g01110) has almost undetectable transcript levels in most tissues analyzed except for immature seeds and green berries (Figure 3). The last step in BR biosynthesis for producing BL is brassinosteroid-6-oxidase 2 (BR6OX2) [61]. In the case of *VviBR6OX2* (VIT_201s0011g00190), its highest expression level was found in seeds, even though it is almost eight times higher in its immature state (Figure 3).

**Figure 4:**
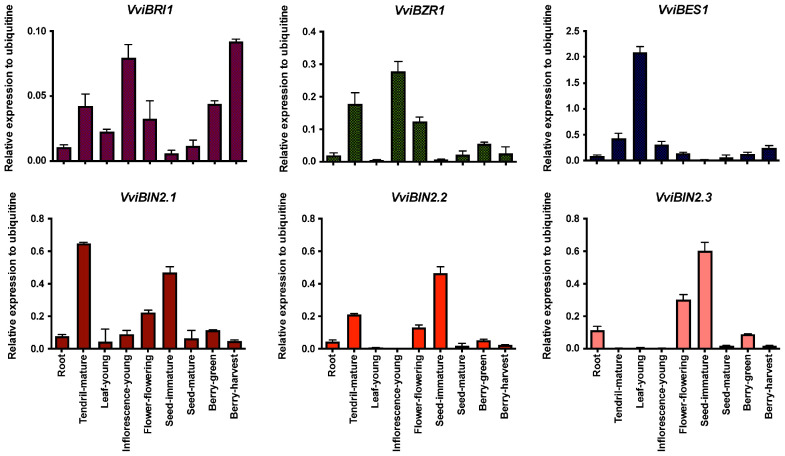
Expression levels of genes involved in brassinosteroid perception and signaling in cv. Cabernet sauvignon grapevines. Expression data was normalized against the reference gene, *VviUBI*. Bars are the mean ± standard deviation of three biological and three technical replicates. Each biological replicate corresponds to a pool of tissue collected from five different plants.

To achieve a more general picture regarding the other pathways, four genes were selected to be analyzed; the canonical BR receptor gene, BRI, genes coding for two transcription factors BZR1 and BES1, and the negative regulator of the signaling pathway BIN2 for which three genic models were found in the genome. BIN2 is a crucial point of connection with other phytohormone signaling pathways, such as auxin and ABA signaling, and is therefore associated with different processes of development, and stress responses [62]. The expression profiles for genes involved in the perception and signaling of BRs were analyzed in different tissues. *VviBRI1* (VIT_207s0031g01850), coding for a brassinosteroid receptor, is ubiquitously expressed in all samples analyzed, while *VviBZR1* (VIT_218s0001g12020) and *VviBES1* (VIT_204s0023g01250), each coding for transcription factors, are primarily expressed in young developing tissues and displayed similarly high levels in inflorescences (Figure 4). Moreover, the highest expression levels of *VviBES1* were found in young leaves, which were twice as high as those of *VviBZR1* in tendrils (Figure 4). *VviBIN2* gene models, which code for key kinases of the signaling pathway, were most highly expressed in tendrils, immature seeds, and flowers at flowering time. Only *VviBIN2.1* (VIT_210s0003g01480) was expressed in almost all tissues with the highest levels of transcript accumulation in mature tendrils, flowers, and immature seeds (Figure 4). Additionally, the expression peaks of *VviBIN2.2* (VIT_212s0028g01810) coincided with those of *VviBIN2.1* in the same tissues; however, they were almost undetectable in young leaves and inflorescences. *VviBIN2.3* (VIT_214s0060g00600) showed a similar pattern of expression with maximum levels in inflorescences, immature seeds, and also in roots (Figure 4).

## 3. Discussion

### 3.1. Endogenous Levels of Brassinosteroids in Grapevines

BRs are sterol-derived phytohormones that regulate growth and stress responses in plants [9,29]. Total sterols in plants comprise 2–3 × 10^−3^ g/g of the plant dry weight while BRs represent only between 10^−12^ to 10^−9^ g/g of fresh weight [6,63]. Compounds belonging to this family of hormones have been isolated from several types of tissues such as seeds, pollen, leaves, flowers, and roots [28]. To date, 62 different natural BRs and 15 precursors have been discovered from different species of plants that include angiosperms, gymnosperms, pteridophytes, and bryophytes [28,64]. Typically, in any given species, between 1 and 24 different detectable BRs are found [64].

In this work, 10 different BRs in grapevine tissues were identified and quantified, a similar number to those found in other dicotyledons species [61]. Of the grapevine BRs, only CS had been previously reported in grape berries [38]. The accumulation of this BR, and that of 6-deoxoCS in berries, was quantified by GC chromatography and assessed to be at the ng/g FW level [38]. The differences found between the previous study [38] and the current research, could be due to the detection method employed (UHPLC-ESI-MS/MS in this work) or the use of fresh weight [38] and dry weight (this work). Most BRs described in our study accumulate below 50 pmol/g DW in grapevines, consistent with the very low levels reported for several species, in which this hormone is found at microgram and nanogram levels per kilogram of fresh weight [64]. The most widely distributed BR in grapevines seems to be HCS, found in all tissues and developmental stages analyzed. The metabolites of the CS group are the most prevalent in grapevines since HCS, 24-epiCS, and CS itself are differentially accumulated in each tissue studied. CS has been described in 53 plants species and is thus the single most widely present BR in the largest number of species to date [64].

It has been described that young tissues accumulate more BRs than mature ones in several species, and usually the highest concentration of BRs have been found in growing tissues such as immature seeds and pollen, and also in reproductive organs [28,65], reflecting their roles in cell division and expansion. Concordantly, different types of BRs showed tissue- and developmental-specific profiles in grapevines. TY is an intermediary in the early C-6 oxidation route and has biological activity itself [66]. The accumulation of TY and CS could indicate that the early C-6 oxidation biosynthesis pathway is active in grapevines. In the case of grapevine seeds, the level of BRs was very low with a maximum of 15.74 pmol/g DW of TY in their mature state. In pea seeds, it has been described that amounts of BL and CS increase rapidly in parallel to weight increases, but then decrease to undetectable levels in mature dried seeds [67]. These results are consistent with intermediary TY accumulation in mature seeds of grapevines, and CS accumulation in green seeds. Moreover, both immature and mature seeds accumulated HCS and 24-epiCS. These compounds were not detected in previous research [67]. Young grapevine tendrils primarily accumulate HCS, while mature tendrils mainly possess DS, 28-norTE, and TY. The intermediate BR, teasterone (TE), is probably rapidly transformed to 3-dihydroteasterone, explaining the low levels found in plants [34]. In our analysis, TE was not detected in any tissue or developmental stage, but 28-norTE was found in several tissues, suggesting that the route for synthesis of 28-nor-BRs is active in grapevines. This route could continue to the synthesis of 28-norCS and/or several intermediates may feed the early C-6 oxidation pathway [35]. Moreover, the quantification of DS suggests that an alternative route using 24-methylene-cholesterol as precursor is also functional in grapevines. The production of CS and 6-deoxoCS in grapevine berries has been previously reported during the ripening stage [38]. Here, additional compounds were found in the different berry developmental stages. Specifically, berries accumulate HDS, TY, 24-epiCS, and DS in different stages of development. At the harvest stage, CS was not detected, unlike in a previous report [38], possibly reflecting experimental differences in the method used for detection. It is interesting to observe the production of high levels of 28-norTE and HCS in mature leaves that could lead to the synthesis of CS, but CS was not found to accumulate in this tissue. This suggests that 28-norTE and HCS are directly used as biologically active BRs.

Grapevine roots mainly accumulate intermediary TY, but DS, HCS, 24-epiBL and DL were also detected. This last BR has been the subject of few studies since its discovery in the legume *Dolichos lablab* [68]. The high levels of TY observed in grapevine roots are consistent with the reports in other species. In tomato, Arabidopsis and pea seedlings, intermediate BRs are mainly found in roots, whilst the active compounds BL and CS, and its precursor 6-deoxoCS are mostly accumulated in leaves [69].

The most bioactive BR and the terminal compound of the biosynthesis route via campesterol is BL [32]. Its C-24 epimer with biological activity, 24-epiBL, was first identified in pollen of *Vicia faba* [70], and it has since been detected in only 10 species, including *Zea mays* L., *Humulus lupulus* L., and *Daucus carota* spp. *sativus* L. [28]. In grapevine samples, BL was not detected in any tissue; however, 24-epiBL reached levels below 1.5 pmol/g DW in roots, paradormant and pre-endodormant buds, rachis at veraison, and berries in late green and veraison stages. The detection of 24-epiBL but not epiBL suggests a rapid synthesis and epimerization of this metabolite.

There is no evidence of long-distance transport of BRs. They have been described as locally synthesized hormones that act in cells adjacent to those of their production [71]. Whether BRs can be transported apoplastically, or whether transporters exist for these phytohormones are open questions. The need to transport some of these hormones away from the site of production could explain the synthesis of large amounts of 28-norTE, HCS, and TY in some tissues analyzed in this work.

Furthermore, BR homeostasis also depends on the rate of conjugation and degradation, meaning that BR content is very variable in time. Processes of BR catabolism have been reported, such as acylation, sulfonation, and glycosylation [72]. A limitation in our research is that we focused only on bioactive molecules. Therefore, it would be exciting to analyze broader spectra of molecules in future studies.

The results obtained from the quantification of BRs in this work allow us a better understanding of their metabolism in grapevines. Together with previously reported results [38], we demonstrate that both the early and late C-6 oxidation pathways are active in grapevines, as well as alternative routes that allow the synthesis of the 28-norBRs or DS compounds. High levels and broad distribution of HCS were discovered in all analyzed tissues, suggesting that this metabolite is the most important BR in grapevines, which may lead to the production of CS in several tissues. As in other species, the synthesis of BRs is a complex process, of which several aspects have yet to be elucidated.

### 3.2. Tissue- and Development-Specific Expression Profile of Genes Involved in the Brassinosteroid Pathway in Grapevines

Unlike other phytohormones, BRs are not transported long distances; rather they act locally at their site of synthesis. So, for maintaining homeostasis, BR regulation in tomato and pea is mainly carried out by transcriptional control of biosynthesis and inactivating genes [73,74]. Expression analysis in Arabidopsis has revealed that each enzyme involved in BR biosynthesis has its own organ- and development-specific profile [61].

The grapevine genes involved in routes of biosynthesis, perception, and signaling analyzed in this work have tissue- and development-dependent expression profiles. CPD/CYP90A1 encodes a cytochrome P450 with 23α-hydroxylase activity [75]. CPD transcript levels are low during the first week of Arabidopsis seedling development and this gene is preferentially expressed in cotyledons and hypocotyls [69], while in grapevines *VviCPD* is mostly expressed in mature tendrils, and mature berries. DET2 encodes a protein with steroid 5α-reductase activity [57]. In the genome of grapevine, two gene models were found for *VviDET2* on different chromosomes, named *VviDET2.1* and *VviDET2.2.* Gene duplication events are characteristic of plant genomes and contribute towards the appearance of novel functions and allow for the improvement of agronomical traits in crops [76]. In Arabidopsis, DWF4/CYP90B1 codes for C-22 hydroxylase involved in the first committed reaction in the biosynthesis of BRs, a proposed flux-determining step [59]. Weak expression restricted to active growing tissues has been reported for this gene in Arabidopsis [58,59]. The analysis of grapevine tissues revealed that *VviDWF4* is expressed in almost all tissues with the exception of the berry in the ripening stage. The highest levels were detected in mature tendrils, young leaves, flowers at flowering, immature seeds and green berries, representing a different expression profile to that found in Arabidopsis. ROT3/CYP90C1 was shown to catalyze C-23 hydroxylation for the conversion of TY to CS in the C6-oxidation pathway of BL [60]. Additionally, this enzyme has been suggested to act as a shortcut from (22S,24R)-22-hydroxy-5α-ergostan-3-one, and 3-epi-6-deoxocathasterone to 3-dehydro-6-deoxoteasterone and 6-deoxotyphasterol, thus bypassing campestanol, 6-deoxocathasterone, and 6-deoxoteasterone [77]. ROT3/CYP90C1 transcripts have been reported in Arabidopsis, especially in roots and in the base of petals and filaments of stamens [69,78]. In grapevines, the pattern was different, with higher expression in mature tendrils, immature seeds, and green berries. CYP90D1 catalyzes C-23 hydroxylation reactions from TE to 3-dehydroteasterone (3DT), and/or 6-deoxoteasterone (6-deoxoTE) to 6-deoxo-3-dehydroteasterone (6-deoxo3DT), or the conversion of cathasterone (CT) to TE, and/or 6-deoxocathasterone (6-deoxoCT) to 6-deoxoTE [60]. CYP90D1 was reported as being preferentially expressed in roots of Arabidopsis [69], consistent with *VviCYP90D1* accumulation in grapevine roots. Moreover, this gene is expressed in immature seeds of grapes. BR6OX1/CYP85A1 encodes for a C-6 oxidase that produces CS [46,61]. Its transcripts are accumulated in roots and hypocotyls of Arabidopsis and also in pea seedlings [67,69,79]. In the case of grapevines, *VviBR6OX1* is accumulated in immature seeds, and green berries, showing a profile associated with reproductive tissues. Finally, BR6OX2/CYP85A2 encodes for a C-6 oxidase that produces either CS or BL [60]. In Arabidopsis seedlings, it is more highly expressed in cotyledons, new leaves and root tips, while in adult plants its profile is associated with reproductive tissues, such as sepals and ovules [79]. The expression analysis of *VviBR6OX2* showed accumulation of transcripts only in seeds, with higher levels in immature states compared to mature ones.

Unravelling the expression profiles of grapevine genes involved in biosynthesis, perception, and signaling of BRs in specific tissues of seedlings and pollen would be interesting considering that high accumulation of this hormone has been reported in other species [28,64]. Despite the participation of BRs in stress processes, we were focused on analyzing BRs in several tissues, but not in stress conditions. Our research lays the basis from which future studies should be performed that consider an analysis of compound accumulation in several tissues over time and during different conditions such as saline and drought stress.

In summary, in this work, metabolites of the BR family and transcripts involved in its biosynthesis, perception and signaling were identified and quantified. It has been demonstrated that P450 enzymes that participate in BR biosynthesis, have a broad substrate specificity, generating multiple intermediate compounds [80]. For this reason, and as found in other angiosperms [28,64] it is difficult to establish correlations between transcript accumulation and metabolite content. As such, additional studies are required, for example dissecting all the metabolites and enzymes of BR biosynthesis pathways, including the alternatives ones. Considering that similar metabolite accumulation patterns and biosynthetic enzymes have been described in several plant species, it has been suggested that BR synthesis is conserved during evolution [81].

In conclusion, the metabolite quantification and gene expression analysis show that different routes of BR biosynthesis are active in grapevines, including canonical and alternative means. Moreover, the accumulation profile suggests HCS is the main bioactive BR in grapevines. Therefore, this work deepens our knowledge regarding BR metabolism and diversity in grapevines and paves the way for analyzing how endogenous and exogenous BRs integrate internal and external cues in the growth and development in this species.

## 4. Materials and Methods

### 4.1. Plant Materials

Grapevine samples (cv. Cabernet sauvignon) were obtained from plants grown in an experimental field located in Curacaví Valley in Chile (33°24′01.0″ S 71°03′17.6″ W) in 2017 and 2018. Phenological stages were determined using the E-L modified system [82]. This is a system that describes 47 numbered states from E-L 1 to E-L 47, where the first state corresponds to a dormant bud (during winter season) and the last state is marked by the end of the fall of leaves during the following autumn. Berry samples were collected approximately every two weeks, from inflorescence in compact state (E-L 15) to harvest (E-L 38). These berry stages sequentially correspond to fruit set (E-L 27), post-fruit set (E-L 29), early green (E-L 32), late green (E-L 33), veraison (E-L 36), and harvest (E-L 38). Moreover, the following tissues were also collected: roots grown in greenhouse conditions two months after germination, young and mature tendrils; young and mature leaves; immature and mature seeds; paradormant and pre-endodormant buds; and rachis in the stages of post-fruit set, pre-veraison, veraison and harvest. In all cases, a pool of tissue from five different plants was collected, frozen in liquid nitrogen and stored at −80 °C until further analysis.

### 4.2. Brassinosteroid Measurements

Brassinosteroid extraction was performed using a protocol previously described [7] with minor changes. For this, approximately 5 mg of lyophilized tissue of grapevines was mixed with 1 mL of extraction solvent (60% acetonitrile) precooled at 4 °C. Zirconium oxide beads of 2 mm were added to the mixture and then homogenized using a vibration mill at 27 Hz for 6 min. Tubes were sonicated for 5 min using an ultrasonic bath and stirred overnight at 4 °C at 17 rpm. The next day, samples were centrifuged for 10 min at 4 °C at 17,000 rpm. The supernatant was transferred to a glass tube (12 × 75 mm) and stored at 4 °C. Centrifugation was repeated to re-extract the pellet and increase efficiency using the previously described protocol. All supernatants were combined and 25 pmol of deuterium-labelled internal standards of BRs was added (Olchemim s.r.o. Olomouc, Czech Republic). After this, brassinosteroids were purified in samples using 50 mg Discovery™ DPA-6S cartridges (Supelco^®^, Bellefonte, PA, USA) and then immunoaffinity (IA) columns (Laboratory of Growth Regulation, Olomouc, Czech Republic). Samples were passed through both columns, vacuum dried, and then resuspended in 50 μL of 100% methanol, charged in vials, and 2 μL injected into a UHPLC-MS/MS system (Waters MS Technologies, Wilmslow, UK). Data was processed using software MassLynx™ version 4.1. (Waters MS Technologies, Wilmslow, UK). Running and detection conditions are identical to those previously described [7].

### 4.3. Bioinformatic Search of Genes in Grapevine Databases and Primer Design

The search for grapevine genes with identity to those described in Arabidopsis was performed using Arabidopsis Information Resource TAIR (www.arabidopsis.org). Sequences obtained from this database were used to query the genomic grapevine database Genoscope (genoscope.cns.fr, accessed on 13 January 2017) for a WEB-BLAT search. From this analysis, information about position in the genome, structure, size, virtual cDNA sequence, predicted protein sequence and ID in Genoscope code was obtained. Using this ID code, the coding sequence (CDS) was acquired from Phytozome (phytozome.jgi.doe.gov, accessed on 15 January 2017). Alignment using the BLASTP tool of NCBI (www.ncbi.nlm.nih.gov, accessed on 16 January 2017) was undertaken to corroborate that gene sequences code for the proteins of interest. BLAST alignments in the grapevine genomic CRIBI database (genomes.cribi.unipd.it/grape, accessed on 20 January 2017) were made to obtain gene IDs in version 2 grapevine format (Supplementary Table S3).

After the identity of the sequences was confirmed, specific real time PCR (qRT-PCR) primers were designed using Primer3 software [83] for the amplification of 100–200 bp of each BR gene. The primers were checked with the IDT Oligo Analyzer program (www.idtdna.com/calc/analyzer, accessed on 27 January 2017) to avoid formation of hairpin structures, homodimers and heterodimers between primers during PCR reactions. Oligos were synthesized by Integrated DNA Technologies Inc. (IDT). The sequences and Tm of primers are shown in Supplementary Table S3.

### 4.4. qRT-PCR Analyses

For qRT-PCR protocols, a SensiMix™ SYBR Hi-ROX commercial kit (Bioline, London, UK) containing ROX as reference dye and SYBR Green I fluorophore was used. Reactions were performed in a Mx3000P real time PCR equipment (Stratagene California, La Jolla, CA, USA). Before measuring the transcripts levels, a standard curve for calculating primer efficiency in each qRT-PCR reaction was undertaken. The accumulation of transcripts of BR genes was determined using three technical and three biological replicas, employing the same program optimized for its standard curve. Each biological replicate corresponds to a pool of tissue collected from five different plants. Normalization of expression was carried out versus *VviUBI,* a gene validated for grapevine in previous studies [84,85]. Relative gene expression was calculated using the ΔΔCt method [86] and visualized with Prism 9 (Graphpad Software Inc, San Diego, CA, USA).

### 4.5. Statistical Analysis

For gene expression data, standard deviation of three biological and three technical replicates were obtained and graphed in Prism 9 (Graphpad Software Inc, San Diego, CA, USA).

## Data Availability

Not applicable.

## Supplementary Materials

The following supporting information can be downloaded at: https://www.mdpi.com/article/10.3390/ijms23031827/s1.

## Abbreviations

24-epiBL	24-epibrassinolide
24-epiCS	24-epicastasterone
28-norTE	28-norteasterone
BES1	BRI1-EMS-SUPPRESSOR1
BIN2	BRASSINOSTEROID INSENSITIVE2
BR6OX	BRASSINOSTEROID-6-OXIDASE
BRI1	BRASSINOSTEROID-INSENSITIVE1
BRs	Brassinosteroids
BZR1	BRASSINAZOLE RESISTANT
CS	Castasterone
CT	Cathasterone
CPD	CONSTITUTIVE PHOTOMORPHOGENIC DWARF
CYP90D1	CYTOCHROME P450 90D1
DET2	DEETIOLATED2
DL	Dolicholide
DS	Dolichosterone
DWF4	DWARF4
HDL	Homodolicholide
HCS	Homocastasterone
HDS	Homodolichosterone
ROT3	ROTUNDIFOLIA 3
TE	Teasterone
TY	Typhasterol
