# Supplementary material for: Characterization of Endogenous Levels of Brassinosteroids and Related Genes in Grapevines
Source: Int J Mol Sci. 2022 Feb 5;23(3):1827. doi: 10.3390/ijms23031827 (PMC8836857; doi:10.3390/ijms23031827)
Supplement: Supplementary file 1 [file ijms-23-01827-s001.zip › suplementary.pdf]

Table S1. Endogenous content of brassinosteroids (pmol/g DW) in different tissues of cv. Cabernet sauvignon grapevines throughout development. Quantification of different brassinosteroid metabolites by UHPLC-MS/MS is shown. Mean ± standard deviation of three biological and three technical replicates are tabulated. Each biological replicate corresponds to a pool of tissue collected from five different plants.

Brassinosteroid metabolite	Root	Young Tendril	Mature Tendril	Young Leaf	Mature Leaf	Paradormant Bud	pre-Endodormant Bud	Inflorescence
Dolichosterone (DS)	2.68 ± 0.83	<LOD	46.97 ± 10.97	<LOD	<LOD	<LOD	<LOD	105.23 ± 20.14
Homodolichosterone (HDS)	<LOD	<LOD	<LOD	<LOD	<LOD	<LOD	<LOD	<LOD
Dolicholide (DL)	0.15 ± 0.01	<LOD	<LOD	<LOD	<LOD	<LOD	<LOD	<LOD
Homodolicholide (HDL)	<LOD	<LOD	<LOD	<LOD	<LOD	<LOD	2.13 ± 0.50	<LOD
28-norteasterone (28-norTE)	<LOD	<LOD	37.59 ± 12.58	34.94 ± 3.50	535.06 ± 63.25	<LOD	<LOD	<LOD
Teasterone (TE)	<LOD	<LOD	<LOD	<LOD	<LOD	<LOD	<LOD	<LOD
Typhasterol (TY)	215.72 ± 51.89	<LOD	33.70 ± 8.95	<LOD	<LOD	24.83 ± 2.69	<LOD	74.09 ± 7.53
Homocastasterone (HCS)	17.84 ± 8.55	18.89 ± 0.45	3.95 ± 0.31	226.21 ± 26.97	455.88 ± 71.07	53.22 ± 9.45	37.65 ± 4.08	42.50 ± 9.42
24-epicastasterone (24-epiCS)	<LOD	9.49 ± 2.82	<LOD	10.75 ± 0.21	<LOD	11.75 ± 0.21	16.40 ± 2.25	<LOD
Castasterone (CS)	<LOD	0.63 ± 0.27	<LOD	<LOD	<LOD	<LOD	<LOD	2.44 ± 0.81
24-epibrassinolide (24-epiBL)	1.05 ± 0.19	<LOD	<LOD	<LOD	<LOD	0.15 ± 0.01	0.30 ± 0.06	<LOD
Brassinolide (BL)	<LOD	<LOD	<LOD	<LOD	<LOD	<LOD	<LOD	<LOD

Table S2. Continuation. Endogenous content of brassinosteroids (pmol/g DW) in different tissues of cv. Cabernet sauvignon grapevines throughout development. Quantification of different brassinosteroid metabolites by UHPLC-MS/MS is shown. Mean ± standard deviation of three biological and three technical replicates are tabulated. Each biological replicate corresponds to a pool of tissue collected from five different plants.

Brassinosteroid metabolite	Rachis at Post fruit set	Rachis at pre-Veraison	Rachis at Veraison	Rachis at Harvest	Immature Seed	Mature Seed	Berry at Fruit Set	Berry at Post-Fruit Set	Early green Berry	Late green Berry	Berry at Veraison	Berry at Harvest
Dolichosterone (DS)	<LOD	<LOD	<LOD	20.42 ± 7.56	<LOD	9.45 ± 1.89	<LOD	<LOD	39.11 ± 5.25	<LOD	52.39 ± 0.51	<LOD
Homodolichosterone (HDS)	<LOD	<LOD	<LOD	<LOD	<LOD	<LOD	49.87 ± 10.94	<LOD	<LOD	<LOD	<LOD	<LOD
Dolicholide (DL)	<LOD	<LOD	<LOD	<LOD	<LOD	<LOD	<LOD	<LOD	<LOD	<LOD	<LOD	<LOD
Homodolicholide (HDL	<LOD	<LOD	<LOD	<LOD	<LOD	<LOD	<LOD	<LOD	<LOD	<LOD	<LOD	<LOD
28-norteasterone (28-norTE)	<LOD	<LOD	<LOD	<LOD	<LOD	<LOD	<LOD	<LOD	<LOD	<LOD	<LOD	<LOD
Teasterone (TE)	<LOD	<LOD	<LOD	<LOD	<LOD	<LOD	<LOD	<LOD	<LOD	<LOD	<LOD	<LOD
Typhasterol (TY)	<LOD	214.53 ± 17.94	146.25 ± 15.21	198.89 ± 11.28	<LOD	15.74 ± 0.86	<LOD	30.76 ± 11.04	1.16 ± 0.61	27.12 ± 10.67	13.97 ± 3.13	11.94 ± 3.74
Homocastasterone (HCS)	5.63 ± 0.87	9.11 ± 0.30	10.44 ± 0.34	21.22 ± 3.92	1.88 ± 0.20	0.70 ± 0.04	5.62 ± 0.75	2.76 ± 0.45	2.37 ± 0.70	4.75 ± 0.60	2.09 ± 0.79	1.72 ± 0.18
24-epicastasterone (24-epiCS)	10.74 ± 0.69	8.69 ± 2.22	9.92 ± 4.51	10.02 ± 1.59	13.06 ± 1.77	12.42 ± 0.84	<LOD	6.09 ± 1.34	56.69 ± 12.70	15.87 ± 3.18	2.17 ± 0.27	7.75 ± 1.91
Castasterone (CS)	0.51 ± 0.02	<LOD	5.56 ± 1.06	<LOD	1.40 ± 0.36	<LOD	5.42 ± 1.14	4.86 ± 1.02	17.40 ± 1.95	9.65 ± 2.43	3.63 ± 0.19	<LOD
24-epibrassinolide (24-epiBL)	<LOD	<LOD	0.54 ± 0.16	<LOD	<LOD	<LOD	<LOD	<LOD	0.09 ± 0.00	0.68 ± 0.29	1.10 ± 0.40	<LOD
Brassinolide (BL)	<LOD	<LOD	<LOD	<LOD	<LOD	<LOD	<LOD	<LOD	<LOD	<LOD	<LOD	<LOD

Table S3. Genic model for putative genes involved in BRs biosynthesis pathway in grapevines. The code (ID), primer sequence, Tm and efficiency of primers for RT-qPCR and size of fragment amplified by PCR are shown.

Gene	ID	Primer sequence (5´-3´)	TM (ºC)	Ef (%)	Size (bp)	Reference
VviCPD	VIT_213s0067g00660	Fw: GACCAGCCGGATCTTACTTATGG<br>Rv: TTCTCAGAGACTCAGTCCACTCC	58	100	107	This work
VviDET2.1	VIT_213s0067g01830	Fw: CACTACAGCTCTTACCATCCATT<br>Rv: CCATAAACCCTTGAGCAATGTG	62	91.7	167	This work
VviDET2.2	VIT_219s0014g00080	Fw: GCCCTTTTCCCAGATGAGAG<br>Rv: GAGATAACAATGGCAGCATCG	62	96	140	This work
VviDWF4	VIT_204s0023g01630	Fw: GAGCTGAAGATGGAAGAGAGGA<br>Rv: AGCAAAGAGCAAGCTCAGTACC	58	99.2	147	This work
VviROT3	VIT_204s0023g02650	Fw: AGGCAGCTAGTGTCAACAACAA<br>Rv: GACGGTGATGGGTAACTTCTTCC	57	98.6	200	This work
VviCYP90D1	VIT_209s0002g02080	Fw: GACTGGCCTTATCAGTTCAACCC<br>Rv: CCACCCACCTGAATTGAGTAACA	57	93.8	166	This work
VviBR6OX1	VIT_214s0083g01110	Fw: GGGGAGACCACTGAGTTTCTTA<br>Rv: TCTAACATGGACTGTGGGTAGC	58	99.8	191	This work
VviBR6OX2	VIT_201s0011g00190	Fw: GATAAGCTGGTGGAAGGAACACT<br>Rv: TAGCCTAACATGTCATCTTGGGTC	58	102	158	This work
VviBRI1	VIT_207s0031g01850	Fw: GGGTCTTTGATTTTTCTGGACC<br>Rv: TGTCCCTTGAAGCCTATTGTAG	58	103.6	192	This work
VviBIN2.1	VIT_210s0003g01480	Fw: GGTCTTGAAGCACTACAGCAAC<br>Rv: TCAAATCCCTATGGCAAACTCC	58	101.9	128	This work
VviBIN2.2	VIT_212s0028g01810	Fw: CCATCAGTTACATGGCAGAGC<br>Rv: TTCAGGGTGACCACATTTGG	62	99.3	181	This work
VviBIN2.3	VIT_214s0060g00600	Fw: CAATAAGTTACATGGCTGAGCG<br>Rv: CTTCAAGGAGACAACATTAGGGT	56	104	182	This work
VviBES1	VIT_204s0023g01250	Fw: CAAGTCCTGTACCCTCTTACCA<br>Rv: CCGCTTTGAACCTCTCGAAGTA	56	100.4	129	This work
VviBZR1	VIT_218s0001g12020	Fw: CTATCGTCTTCCAAAACACTGCG<br>Rv: GGAAGTTCCTGCAATCTCAGTT	56	97.9	142	This work
VviUBI	VIT_216s0098g01190	Fw: TCTGAGGCTTCGTGGTGGTA<br>Rv: AGGCGTGCATAACATTTGCG	55	99	99	[48,49]

Table S4. **CDS FASTA sequences for gene selected coding for BRs pathways in grapevines.**

> *VviCPD*_VIT_213s0067g00660
ATGAAGGGTAATCTCCACAAAAAAATGCATTCCCTCACCATGAGTTTCGGTAATTCTTCCATCATCAAG
GACCACTTGCTGCTCGACATCGACCGCTTGATCCGCTTCAACATGGACTCATGGACCAGCCGGATCTTA
CTTATGGAAGAGGCCAAGAAGATAACATTCGATTTGACAGTGAAGCAGCTGATGAGCTTTGATCCAGG
GGAGTGGACTGAGTCTCTGAGAAAAGAATATGTGCTTGTGATTGAAGGCTTCTTTACCGTCCCTTTCCC
TCTCTTCTCCGCCACCTACCGCAGAGCCATCCAAGCAAGGACGAAGGTAGCAGAGGCACTGAACTTGG
TAGTGAGGGAAAGGAGGAAAGCCAAGGAAGAAGGAGAAGAGGAGAAGAAGAACGACATGCTGGCC
GCCCTCCTCGACAGCGGCGATAACTTCTCCGACGAGCAGATCGTGGATTTCATCCTGGCCCTGCTGGTG
GCTGGCTACGAGACCACCTCCACCATCATGACCCTCGCGATCAAGTTCCTCACAGAGACCCCTCTCGC
CTTGGCTCAGCTCAGGGAGGAGCATGACGAGATTAGGGCAAGGATAAGTGACCCTAATGTTCTGGAAT
GGAGTGATTATAAATCCATGCCTTTCACTCAATGTGTTGTGAATGAGACTTTGAGGGTGGCAAACATA
ATAGGTGGGATTTTCAGGCGAGTAACCACTGACATCCATGTCAAAGGTTACACAATTCCTAAGGGATG
GAAGGTGTTTGCATCATTTCGAGCTGTACACCTGGACCATGAATACTTCAAGGATGCTCGTACTTTTAA
TCCCTGGAGATGGCAGAATAATTCTGGACCCACGGTTCCAGGGAGTGGTGTGAATGTGTTCACACCAT
TTGGAGGAGGGCCGCGCCTGTGCCCTGGCTATGAGCTTGCAAGGGTGGTGCTCTCGGTTTTCCTTCACC
GCATGGTCACTCGATACAGCTGGGCTCCTGCAGAGGAGGATAAGTTGGTGTTCTTTCCAACCACTAGG
ACTCAGAAGCGATACCCCATCAATGTGGAGAGGCGAAAAGTGTCATCCGCCGCAGAAAAAGGTGAGG
GATGA

> *VviDET2.1*_VIT_213s0067g01830
ATGGGAATTAATCTTCAGTATTCCAAATTATGGAATCCTAATTCCAAGAGAATCAAAGTCTCCAGCAG
AACGGGGATGCTTCTACTGTACACTCCGGCCTTTCTGGTGGGTTTTGCTTCCTTTTGGATTTTCCCAGAA
AAAGGTTTCCGACCTCTGTTGGTCACTACAGCTCTTACCATCCATTTCTTCAAGAGAGTTTTGGAGGTG
CTGTTTGTACACAAATACAGTGGTGGCATGGTCCTGGATTCTATCATTCTCATCTCTTCTGGCTATTCTA
CCACCACTGCAACCATGATCCATGCCCAACACATTGCTCAAGGGTTTATGGAGCCAGCAATTGATCTC
AAATGCCCTGGGATTCTACTGTTTTCAGTGGGAATTTGTGGTAATTTTTACCACCACTACCTCCTTTCCA
AACTAAGGGGAAAAGGTGAGAAAGGCTACAAAATTCCAAAAGGGGGTCTCTTTCACCAAGTTATATG
CCCACACTATCTTTTTGAGATTCTAGAGTTTGTTGGGGTATTCTTCATCTCTCAAACACTCTATTCATTA
TCCTTCACAATAAGCACAACTTTGTACTTGATTGGAAGGAGTTATGCTACTAGGAAATGGTACATATCC
AAATTTGAGAATTTCCCTAGGGAGACCAAGGCTCTAATTCCATACATTTTCTAG

> *VviDET2.2*_VIT_219s0014g00080
ATGTCAGTGGTGAGTTTTGCATCAATGGCAAATGCAGGGCTATCAGAGTTGAAAGGAAAACACATGG
AATACTCCAAGTTCTGGGAGCTCAAGTCTGGGAGAAAGTACAAAGTGGATAGTAGAACAGGCATGAT
CGTGTGTTATACTCCAGCATTTCTTGCTGGTGTGGCCTCTTTTGCCCTTTTCCCAGATGAGAGTTTCAGG
TTTTTGCTGCTCACTTCGGCTCTCTCCATCCACTTCTTCAAGAGGATATTTGAGGTGCTATTTGTTCACA
AATACAGTGGTGGGATGGCTCTCGATGCTGCCATTGTTATCTCTCTTAGCTACTTCATCTCTACTGCAA
CTATGATCTATGCCCAACACCTCTCTCGAGATTTTCCGGAGCCGCTAATGGATCTCAAGTATCCAGGAA
TGGCCAAGGGTGAGAGAGAGTATAAGATTCCCAAGGGCACAGCCTTGTATTTGATGGGAAGAAGTTA
TTCTACTAGGAGATGGTATGTTTCCAAGTTTGAAAACTTTCCCAATAATGTCAAGGCGCTTATTCCATA
CATTCTCTAA

> *VviDWF4*_ VIT_204s0023g01630
ATGTCTGACTTGGAGTTTTTTCTCCTCCTCCTTGCGTCAATCTCAGTTCCCCTTGTGATTCTCAATCTGA
TCAAAAGAAAGCAAAGCGGACTTAATCTACCTCCGGGAAACACGGGTTGGCCTTTTCTGGGTGAAACC
ATCGGCTATTTAAAGCCGTACTCGGCCACTTCAATAGGAGACTTCATGGAGCAACACATATCAAGGTT
CGGAGAAATCTACAAGTCCAATCTGTTTGGCGAGCCAACCATAGTCTCAGCGGATTCTGGGCTGAACA
GATTCATACTACAGAACGAAGGAAAATTGTTTGAATGCAGCTATCCCAGAAGCATAGGTGGAATTCTT
GGGAAATGGTCCATGCTGGTTTTAGTTGGAGACATGCATAGAGACATGAGAACCATCTCCCTCAACTT
CTTGAGCCATGGCAGGCTTAGGACTCATCTCCTACCAGAGGTGGTGAAGCACACTTTGCTTGTTCTAAG
CTCTTGGAAGGAGAATTGTACATTTTCTGCTCAAGATGAAGCTAAGAAGTTTACCTTCAATCTGATGGC
AAAACATATCATGAGCTTGGATCCCGGAAAGCCGGAGACTGAGCAGCTTAAGAAAGAGTATGTTACTT
TCATGAAAGGAGTGGTATCTGCTCCTTTGAATTTCCCTGGAACTGCATACAGAAAAGCCCTACAGTCTC
GGTCCACCATCTTAAAATTTATCGAGCTGAAGATGGAAGAGAGGATTCAGAAACTGAGGGGAGGAGG

GTTTGAGAATATGGAGGATGACGATCTTCTTGGATGGGTGCTGAAGCATTCCAACCTTTCAACTGAGC
AAATCCTGGACTTGGTACTGAGCTTGCTCTTTGCTGGCCATGAAACTTCATCAGTAGCAATAGCTTTAG
CTATCTACTTCTTGGAAGGCTGTCCCAACGCCGTTCAACAGCTAAGAGAAGAACACCTTGAAATTTCC
AGGGCCAAGAAGCAATCAGGAGAGTTGGAATTGAGTTGGGAAGACTACAAAAAAATGGAATTCACTC
AATGTGTTATTAGTGAGACACTGAGGCTTGGAAATGTGGTTAGGTTTCTACACAGAAAGGCCCTGAAA
GATGTCCGGTACAAAGGGTATGACATTCCATGTGGGTGGAAAGTGCTTCCGGTGATTGCAGCAGTGCA
CTTGGATCCTTCCCTTTTCGACCAACCTCAACACTTCAATCCTTGGAGATGGCAGCAGAATAATGGTAA
CCGAGGAAATTCGACTAGCTGGAGCACAGCCACGAATCAAAACTTCATGCCATTTGGGGGAGGACCA
CGGCTATGTGCGGGCTCTGAATTGGCCAAACTGGAAATGGCGGTGTTCATCCACCATTTGGTTCTCAAC
TACAACTGGGAGTTGGTTGATAAGGATCAGGCTTTTGCCTTCCCATTCGTCGATTTTCCGAAGGGCCTA
CCCATCAAGGTCCGACACCACAATTTCACGTGA

> *VviROT3_* VIT_204s0023g02650
ATGGAGGTGATTCATTGGTGTTGGGTTGTGGTGGGTGTTGTGATGGGGTGGTGTTGGTATAAGAGCAT
CAAGAACAAGGCGAAGGAGGAGGGAAGTGGGGTTCCCAGAGGAAACCCTGGTTGGCCAGTAATAGG
AGAGACTCTTGACTTCATTGCTTCTGGTTGGAGCTCTCGACCAGTCAGCTTCATGGAGAAGCGCAAGTC
TCGATATGGGAAGGTGTTCAAGACAAACATATTGGGAAAGCCGGTGATAGTGTCGACGGATCCGGAG
GTGAACAAGGTGGTTCTGCAGAACATTGGGAATGTATTTATCCCTGCCTATCCCCAAACCATCACGGA
GATACTTGGAGAATCATCTATACTCCAGATGAACGGCAGTCTGCAGAAGAGAGTGCATGCACTGCTTG
GTGGCTTCTTGAGATCACCACAACTCAAAGCTCGAATCACCCGTGACATTGAACGCTATGTCAAACTC
ACCCTTGATTCTTGGAAAGACCAGCACATTGTTTACGTCCAAGATGAAGTCAGAAAGATCACGTTTGA
CGTTCTGGTTAGAGTTCTGGTGAGCATTACTCCAGGTGAAGATTTGAACTTTATGAAGAGAGAATTCG
CAGAAGTCATCAAAGGCTTGATTTGTCTCCCAATTAAACTTCCCGGAACTAGACTATATAAATCTCTCA
AGGCTAAGGAGAGGCTGTTGAAGCTGGTGAGAAGGATCGTGGAGGAGAGGAAGGCAGCCATGGATA
GAGGTGATCATGAGACCCTCAAGGGTCCACCCAACGATGCCGTGGACGTGCTGCTCCGTGACACAGGG
GATTCATCTGAGACGTCGCGTCTGTCGTTGGATTTCATCAGTTCGAATATGATCGAGATGATGATCCCC
GGGGAGGAGACTCTGCCGACCGCCATGACCCTGGCCGTCAAATTTCTAAGTGACTGCCCCGTCGCTCT
GAACCAGCTGATGGAGGAGAACATGGAGTTAAAGAAGGGAAAGGCTCTTTCCGGGGAGGATTATGCC
TGGACTGACTACATGTGCTTGCCATTTACTCAAAATGTGATCAATGAAACTCTGAGAATGGCAAATAT
CATAAATGCGGTTTGGAGAAAAGCTGTTAAAGATGTCAAAATCAAAGATTACCTGATACCAGAGGGA
TGGGGTGTCATGGCATCTTTCACTTCCATTCACATGGATGAAGAGAATTATGAAAACCCTTATCAGTTC
AATCCATGGAGATGGGAGAAGGCAGCTAGTGTCAACAACAACAATAGCTATACACCCTTCGGAGGTG
GACAGAGGCTGTGCCCTGGTTTAGAATTATCGAAACTCGAAATCTCAATTTTTCTTCATCATCTCGTCA
CCACCTACCGGTGGGTGGCTAAGAAGGACGACGTAGTTTACTTTCCAACAGTGAAGATGAGGAAGAA
GTTACCCATCACCGTCACACCCTTACCACCCCTTTGA

> *VviCYP90D1_* VIT_209s0002g02080
ATGCACAACTTGTGGATTGTGTTTTTGACAGCCATCTTCTTGTCCACCATAATTCTTCTCTACAGAAAC
AGATCTAGAATCAGATCATCACCAAGCTCATCACTTCCTCTAGGAACTCTTGGATGGCCTCTTATCGGC
GAAACCCTAGAGTTCATCTCTTGTGCCTACTCAGACCGCCCTGAGAGCTTCATGGAGCGGCGGCGTCG
CATGTATGGAAAGGTGTTCAAGTCTCACATATTTGGGAGCCCAACAATAGTTTCCATTGATGCAGAAG
TGAGTAGATTTGTGTTGCAGAGTGATTCAAAGGCCTTTGTGCCATCTTACCCTAAATCTCTCACTGAGC
TGATGGGGCAGTCCTCCATTTTGCTCATCAATGGAAGCTTGCAGAGGAGAGTCCATGGACTGATTGGA
GCCTTCTTCAAATCCCCACATCTCAAAGCTCAAATCACTCAAGAAATGGAGAGTTATATTCAGAAATC
AATGGGGTCTTGGAGAGATGATCACCCCATTTTCATCCAGGATGAAGCAAAAAATATTGCTTTTCAAG
TCCTAGTGAAGGCACTGATCAGTTTGAATCCAGGTGAAGAAATGGAGTTTCTAAGAAAACAGTTCCAG
GAATTCATCTCAGGCCTCATGTCCTTGCCTGTAAACATACCCGGAACAAGGCTTTACAGATCTTTACAG
GCCAAGAAGAAAATGGTTCAATTAGTAGGAAAAATCATCCAAGAAAGGAGAAATATCAACCAACCCT
CCAAGGTCCCCAAAGATGTTCTGGATGTTCTCCTCAATGACTCAAGCCAACTACTGACCGATACCCTCA
TATCCGATAATATGATCGATCTGATGATTCCTGGAGAGGATTCAGTACCAGTTCTCGTGACTCTCGCGA
TCAAATACCTCTCCGATTGCCCTGCAGCCCTCCAACAACTGACGGAGGAGAACATGAGACTGAAGAGA
CTAAAAGCCGAGCGGGGAGAGACGATGACTTGGAGCGATTACTTATCGCTACCATTCACGCAGACGGT
GATCACAGAAACACTAAGGTTGGGAAACGTTATTATAGGGGTGATGAGGAAGGCCATGAAGGATGTG
GAGATAAAGGGACATAGGATACCAAAAGGATGGTGCGTTTTCGCATATTTTCGGTCTGTCCATCTTGA
TGAAAGCCAGTATGACTGGCCTTATCAGTTCAACCCATGGAGGTGGCAAGACAAAAATATTAGCAGTT
GTAGCTTCACTCCTTTTGGTGGTGGACAAAGGCTGTGTCCAGGGCTTGACTTGGCCAGGCTGGAGGCTT

CAATCTTCCTCCACCACTTTGTTACTCAATTCAGGTGGGTGGCTGAGGATGATTCAATTGTCAACTTCC
CCACAGTAAGAATGAAGAGAAGGATGCCTGTTTGGGTCAAAAGGAGAAGAGACTACTCTTGA

> *VviBR6OX1_* VIT_214s0083g01110
ATGGCTGTTTTCATGGCGGTTTTTGGTGTGGTTCTTATTGGGCTCTGTATTTGCACTGCTTTGTTGAGAT
GGAATGAGGTGAGGTACAGGAAGAAAGGATTGCCCCCAGGTACAATGGGTTGGCCAGTCTTTGGGGA
GACCACTGAGTTTCTTAAGCAAGGCCCAAGCTTCATGAAAAACCAGAGAGCAAGGTATGGAAAATTTT
TCAAATCCCATTTATTGGGTTGTCCTACCACAGTCTCCATGGATCCAGAGCTCAACAGATACATCCTAA
TGAATGAAGCAAAAGGCCTGGTTCCTGGCTACCCACAGTCCATGTTAGATATCCTGGGAAAATGCAAC
ATTGCAGCTGTCCATGGCTCCACTCACAAGTACATGAGAGGGGCACTTCTAGCTCTCATTAGCCCCACC
ATGATAAGAGGCCAGCTTTTACCAAAGATAGATGAGTTCATGAGATCCCATCTGAACAAATGGGATAC
CAAAATCATCAACATCCAGGAGAAAACCAAAGAGATGGCACTTCTCTCTTCTCTCAAGCAGATTGCCG
GTATTGAATCCGGCACAATATCTAAGGAGTTTATGCCAGAGTTTTTCAAGCTGGTGTTGGGAACCATTT
CTCTGCCTATTGACCTTCCTGGCACAAATTACCGCCGCGGGTTCCAAGCAAGGAAGAATATTGTTGGC
ATGTTGAGACAACTCATAGAGGAGAGAAAAGCTTCCCAGGAAACCCACAATGACATGCTTGGTTGCCT
AATGAGAACTAATGAAAATCGATATAAACTAAGCGATGAAGAGATAATTGATCTCATAATCACAATTC
TGTATTCGGGTTACGAGACTGTTTCCACCACTTCCATGATGGCTGTCAAGTATCTCCATGACCACCCAA
GAGTTCTTGATGAACTCAGAAAAGAACATTTGGCAATCAGGGAAAGGAAAAGGCCAGAGGATCCCAT
TGACTGGAATGACTACAAGTTGATGCGCTTCACCCGAGCTGTGATCTTTGAGACCTCCAGACTGGCTA
CAATTGTTAATGGGGTTTTGAGGAAAACCACCAAAGACATGGAACTGAATGGATTTGTGATTCCAAAA
GGCTGGAGAATATATGTTTATACCAGGGAGATCAACTATGACCCCTTGCTATACCCCGACCCGTTAGC
TTTCAACCCTTGGAGATGGCTGGACAAGAGCTTAGAGTCCCAAAACTACTTCCTACTATTTGGAGGAG
GCACCAGGCAGTGTCCCGGAAAGGAACTGGGAATAGCTGAAATTTCTACATTCCTTCACTACTTTGTA
ACAAGATACAGATGGGAAGAAGTTGGGGGAGATAAGCTAATGAAGTTTCCAAGAGTTGAAGCACCAA
ATGGACTACACATTAGGGTCTCAGCTTACTGA

> *VviBR6OX2_* VIT_201s0011g00190
ATGGCTGTTTTGGTGGTGATCTTTGTTTTGGTTGTGGCTTTATCTGTCTGCTTTGCTCTCTTGAAATGGA
ATGAGATAAGATACAGCAGGAGAGGGCTGCCTCCTGGGACGATGGGGTGGCCACTCTTTGGTGAGAC
TACTGACTTCATTAAACAGGGACCTGACTTCATGAAGAAGCAGAGAGCAAGGTACGGGAGCTTTTTCA
AGACCCACATACTGGGCTGTCCTACCATTATTTCCATGGATCCAGAGCTCAACAGATACGTCCTTTTGA
ATGAAGGAAAAGGACTTGTTCCTGGCTACCCACAGTCCATGTTGGATATATTAGGGGAACACAACATT
GCTGCTGTACAGGGATCGACCCACAAATATATCAGAGGGTCAATGCTATCCCTCATTGCCCCCCCCAT
GATCAAAGATCAGCTTCTCAGAAAGATTGATCAGGGCATGAGATTCCACCTCAGCAACTGGGATGGCA
GAACCATTGATATCCAAGAAAAAACCAATGAGATGGCACTGTTCATTCCCTTCAAGTTGATCATGGAA
ACTGAATCAGCCTCCATCTATGAGACCTTCAAAAGGGAGTTTGATAAGCTGGTGGAAGGAACACTGTC
ACTGCCCATCAACATTCCTGGCACAAGTTACCATCATGGGTTCCAGGGAAGGAAGAATGTTATTAGAA
TGTTGAAAGGAGTTATGGAGAAGAGAAGAGCTTCTTCAATGACCCAAGATGACATGTTAGGCTACCTT
CTTAGGAATGAAGGATCAAAGTATAATCTCAGTGATGAGGAGATACTTGATCAAGTAATAACAATTTT
GTATTCAGGATATGAAACTGTCTCAACTACTTCCATGATGGCCGTCAAATATCTGCTTGATAATCCGAG
AGCTCTTCAACAACTTAGAGAAGAGCATTTAGCGATTCGACAAAGAAAAAATCCAGAGGATCCAATT
GACTGGAACGATTACAAGTCCATGAACTTTACTCGAGCCGTGATCTTTGAAACCTCGAGGTTGGCCAC
AGTCGTGAATGGGGTGCTGAGGAAGACAACAAAAGAAATGGAGTTGAATGGATTTGTGATTCCTAGA
GGGTGGAGGATATATGTTTACACAAGGGAGATCAACTATGATCCTTTCCTCTATCCAGAGCCATATAC
CTTCAATCCATGGAGATGGCTGGACAACAGCTTGGAATCTCACAATTACTGCTTCACATTTGGAGGTG
GAACCAGGCTCTGTCCCGGAAAAGAATTGGGCATAGTTCAGATTTCTACATTCCTTCACTACTTCCTTA
CTAGTTATAGGTGGGAAGAAGTGGGGAGTAACAAGATTGTGAAATTCCCAAGAGTAGAGGCACCAAA
TGGCCTGCATATAAGGGTGTCCAAGTACTAA

> *VviBRI1*_VIT_207s0031g01850
ATGGCATGTGAGCTACAGCTCACCGGTTTCAGAGGACCCAAGATGAAAGCTTTTTCTCGCTCTTACTCT
CAGACCTACTTGTTTTTCTTGTTTTTGTTCATGAGTTTTTTGTGTGTGGCTTTAGCGGCGGTTTCCAAGG
ATGCGACGCTGTTGTTGAGCTTCAAGAGGTCGCTGCCCAATCCTGGTGTGCTTCAGAACTGGGAGGAG
GGCAGAGACCCATGTTACTTCACTGGAGTTACGTGCAAGGGCGGGCGAGTATCGTCTCTGGATCTCAC
CAGCGTGGAGTTGAATGCGGAGCTTCGATACGTGGCTACATTCCTCATGGGGATTGACCGGTTGGAGT
TTCTCTCTCTGCAATCCACCAACCTCACTGGAGCTGTCTCGTCGGTTTCCGGGTCTCGTTGCGGCGCGTT

GCTGAGCTCCCTGGATCTTGCCAACAACACCGTATCTGGGTCGATCTCGGATTTGGAAAATCTGGTGTC
GTGCTCTTCTTTGAAGTCTCTGAACCTGTCCAGGAACAATCTGGAGTTCACCGCCGGAAGGCGGGATT
CCGGCGGGGTGTTCACCGGTTTGGAGGTGCTGGACCTGTCGAACAACCGTATATCCGGCGAGAATGTG
GTGGGGTGGATTTTATCCGGCGGGTGTCGTCAGCTGAAAAGCTTGGCCTTGAAGGGGAACAACGCCAA
TGGAAGTATACCACTCTCTGGGTGTGGAAATTTGGAGTATTTGGACGTTTCTTTCAATAATTTCTCTGC
GTTTCCATCGCTGGGTCGTTGCTCCGCTCTCAACTATCTGGATCTCTCCGCCAATAAATTTTCCGGCGA
GATTAAGAACCAGCTCGCGTACTGTCAGCAGCTCAACCACCTCAATCTCTCCAGCAACCACTTCACCG
GCGCGATTCCGGCGCTGCCCACTGCTAATTTGGAGTACGTTTATCTCTCCGGCAACGATTTCCAGGGTG
GCATACCTCTGCTTCTTGCCGACGCCTGTCCCACTCTCCTCGAGCTCAACCTCTCCTCCAACAACCTCTC
CGGTACGGTCCCGAGCAACTTCCAGTCGTGTTCTTCTCTGGTATCCATTGATATATCTAGAAACAACTT
CTCCGGCGTTTTGCCGATTGATACTCTTTTGAAATGGACCAATTTGAGGAAGCTCTCTTTGTCTTACAA
CAATTTTGTCGGTTCTTTGCCGGAATCTCTGTCCAAGTTGATGAATTTGGAGACTTTGGATGTGAGTTC
CAACAATTTTTCGGGTTTGATCCCTTCCGGACTGTGTGGAGATCCCAGGAACAGCCTGAAGGAGCTTC
ATCTTCAGAACAATTTGTTCACTGGTCGTATACCAGAGGCTCTGAGCAACTGTTCTCAGCTGGTCTCCC
TTGATCTCAGCTTCAATTACCTCACAGGGACGATCCCCTCCAGCCTGGGCTCCTTAACTAAGCTCCAGC
ATTTGATGCTGTGGCTGAATCAGCTCCATGGACAGATCCCAGAGGAGCTCATGAACCTCAAAACCCTC
GAGAATCTGATTCTAGACTTTAACGAATTGACCGGCCCCATCCCTGACGGCCTTAGCAACTGCACCAA
TTTGAATTGGATCTCCCTTTCAAACAACAGGTTGAGTGGTGAGATTCCCGGGTGGATCGGTAAGCTATC
CAATCTTGCCATCCTCAAGCTCGGGAACAATTCATTCTACGGTAGCATTCCGCCGGAGCTTGGGGATTG
TCGCAGTTTGATATGGTTGGATCTCAATACTAATCACTTGACTGGCACCATCCCGCCCGCCCTGTTCAA
ACAGTCTGGTAATATCGCTGTTGGGCTGGTGACAGGGAAGAGCTATGTGTATATTAGGAATGATGGGA
GTAAGGAGTGCCATGGAGCTGGGAATTTGCTGGAGTATGGGGGAATCAGAGAGGAGGAGATGGACCG
TATTTCGACTAGGAATCCATGCAACTTCACTAGAGTCTACAAGGGTAGGACAAATCCCACATTCAATC
ATAACGGGTCTTTGATTTTTCTGGACCTTTCATATAACATGTTGGGAGGTAGCATTCCAAAGGAGTTGG
GGACTCCTTATTATCTGTATATTCTGAATTTGGCTCATAACAATCTCTCTGGTGCCATTCCTGTGGAGCT
TGGAGGCTTGAAGAATGTTAATATTCTTGATTTCTCCTACAATAGGCTTCAAGGGACAATTCCACAATC
TCTGTCTGGCCTTTCCATGCTGAATGACATTGATCTGTCTAACAACAATCTATCTGGGACGATTCCTCA
ATCAGGTCAATTCTTAACGTTTCCGAATTTGAGCTTCGCTAACAATTCTGGCCTCTGTGGGTTTCCCCTT
TCCCCCTGTGGGGGAGGTCCCAACTCGATTTCAAGTACCCAGCATCAGAAATCTCATCGGAGACAAGC
ATCCCTTGTGGGGAGTGTGGCCATGGGCTTATTGTTCTCCCTGTTTTGCATATTTGGTTTGATCATAGTT
GCCATTGAGACGAGAAAAAGGAGGAAAAAGAAGGATTCTACTCTTGACGTTTATATTGATAGTAATTC
CCATTCGGGCACTGCAAATGTTAGCTGGAAGCTAACTGGCGCCCGGGAAGCATTGAGCATCAACCTAG
CCACATTTGAAAAACCCCTTCGGAAGCTCACCTTTGCTGATCTTCTTGAAGCCACCAATGGCTTCCACA
ATGACAGCCTTATTGGCTCTGGTGGCTTTGGCGATGTGTACAGAGCTCAGCTGAAAGATGGAAGCATA
GTAGCCATCAAGAAGCTGATACATATAAGCGGACAGGGGGACCGGGAATTCACGGCTGAAATGGAAA
CCATTGGGAAAATCAAGCACCGAAACCTCGTCCCTCTCTTGGGCTACTGCAAGGTGGGGGAGGAACGG
CTCTTGGTGTATGAATACATGAGGTTTGGAAGCCTGGAAGACATCCTACATGACCGGAAAAAGGCTGG
GATCAAGCTGAATTGGGCAGCTAGGAGGAAGATTGCCATTGGAGCTGCAAGGGGATTAGCCTTCCTCC
ACCATAACTGCATCCCCCACATCATCCACCGCGACATGAAATCGAGCAACGTCCTGCTTGATGAAAAC
TTTGAAGCCAGAGTCTCTGACTTCGGAATGGCTAGGCTAATGAGCGCAATGGACACCCATTTGAGTGT
CAGCACGCTTGCAGGCACCCCCGGCTATGTCCCTCCCGAGTACTACCAGAGCTTTAGATGTTCTACAA
AAGGCGATGTCTACAGTTATGGAGTAGTTCTGCTAGAGCTTCTAACAGGGAAGCAACCAACAGATTCA
GCTGATTTTGGGGATAACAACCTTGTGGGGTGGGTGAAGCAGCATGCCAAACTGAGAATAAGCGATGT
ATTCGATCCTGAGCTCATGAAGGAGGACCCCAACCTGGAGATTGAGCTCTTGCAACACTTAAAGGTAG
CGTGTGCCTGTTTGGACGATCGGCCATGGCGGCGGCCCACCATGATTCAAGTCATGGCGATGTTCAAG
GAGATCCAAGCAGGGTCTGGGCTGGACTCTGCATCAACCATAGCCACCGAGGATGGAGGCTTCAGTGC
AGTGGAAATGGTAGAAATGAGCATAAAAGAAGTCCCGGAATTCAGCAAGCAGTAG

> *VviBIN2.1_* VIT_210s0003g01480
ATGTCCGACGACAAGGAAATCTCTGCCCCTGTTGTTGATGGGAATGATCCTGTCACAGGTCACATCATT
TCCACCACAATTGGAGGGAAAAATGGGGAGCCCAAACAGACCATCAGTTACATGGCAGAGCGTGTTG
TGGGTACCGGGTCATTTGGAATTGTTTTTCAGGCAAAATGCTTGGAAACTGGAGAGACTGTGGCCATA
AAGAAGGTTTTGCAGGACAGAAGATATAAGAATCGTGAGCTGCAGTTAATGCGCACGATGGATCATC
CAAATGTCATTTCCCTAAAACATTGTTTCTTTTCTACTACAAGTAGAGATGAACTTTTCCTCAACTTGGT
AATGGAATATGTCCCTGAGACCATGTATCGGGTCTTGAAGCACTACAGCAACGCAAAACAGCGAATGC
CTCTTATATATGTGAAACTTTACACTTACCAAATTTTCAGGGGCCTGGCATATATCCACAGTGTTCCTG

GAGTTTGCCATAGGGATTTGAAGCCTCAAAATCTTCTAGTTGATCCACTTACTCACCAAGTTAAGCTTT
GTGACTTTGGAAGTGCAAAAGTGCTAGTTAAAGGTGAAGCAAACATATCATACATATGTTCTCGTTTC
TACCGAGCACCTGAACTCATATTTGGGGCCACAGAGTATACAACCTCAATTGATATATGGTCAGCTGG
TTGTGTCCTAGCTGAGCTTCTTCTAGGCCAGCCATTGTTTCCTGGAGAGAATGCAGTGGACCAGCTTGT
TGAGATTATCAAGGTTCTTGGTACACCAACGCGGGAAGAAATTCGCTGTATGAATCCGAGTTATACTG
ATTTTAGGTTTCCACAGATAAAAGCACACCCTTGGCACAAGGTCTTTCACAAACGAATGCCTCCTGAA
GCAATCGATCTTGCTTCTCGACTTCTGCAATACTCACCAAGTCTTCGCTGCACAGCTCTAGAAGCATGT
GCACATCCTTTCTTTGATGAGCTCCGAGAACCCAATGCCCGCCTGCCAAATGGTCGTCCATTACCACCT
CTCTTCAATTTTAAGCAGGAATTATCTGGGGCTTCTCCTGAGCTTGTTAATAAGTTGATACCGGAGCAT
GTGAGGAGGCAAATAGGCCTAAGTTTTTCCACCCAGCAGGCACATAA

> *VviBIN2.2_* VIT_212s0028g01810
ATGGCCTCGCTGCCTCTGGGTCCTCAAACCCACCACCACCACCACCACCTTCCGCCGGAACACGACCC
TCTCAAACCTCCTTTCCGCCGCCCCGACCACCTCGACTCCGACAAGGAAATGTCAGCTGCTGTTATTGA
GGGGAATGATCCAGTCACTGGTCATATAATTTCCACCACCATTGGAGGCAAAAATGGAGAGCCTAAAC
AGACCATCAGTTACATGGCAGAGCGCATTGTAGGCACTGGATCGTTTGGAATTGTTTTTCAGGCAAAA
TGCTTGGAAACTGGAGAAACTGTCGCAATAAAAAAGGTCTTACAAGACAGGCGGTATAAAAATCGTG
AGCTGCAGTTATTGCGCTTGATGGATCACCCAAATGTGGTCACCCTGAAGCACTGCTTCTTTTCCACAA
CAAGTAGAGATGAACTTTTTCTGAATTTGGTAATGGAATATGTTCCTGAGACTATGTACCGGGTCTTGA
AGCATTATAGCAATGCTAACCAGAGGATGCCTCTCATATATGTCAAACTTTATACATATCAAATTTTTA
GGGGGCTGGCCTATATTCATACAGTGCCTGGGGTTTGCCATAGGGATGTGAAGCCTCAAAATGTTCTG
GTTGATCCTCTCACCCATCAGGTTAAGCTTTGTGATTTTGGAAGTGCAAAGGTTCTGGTCAAGGGTGAA
GCAAACATATCATACATTTGTTCTCGCTACTACCGGGCCCCAGAACTTATATTTGGTGCAACTGAATAC
ACAACATCGATTGATATTTGGTCAGCTGGTTGTGTTTTTGCTGAGCTTCTACTAGGCCAGCCCTTGTTTC
CTGGAGAGAGTGCAGTAGACCAACTTGTTGAAATTATAAAGGTTCTTGGTACTCCAACTAGAGAAGAA
ATTCGATGTATGAACCCGAATTATACTGATTTCAGGTTTCCTCAGATCAAAGCTCATCCTTGGCACAAG
GTGTTTCACAAACGGATGCCTCCTGAAGCTCTTGATCTTGCTTCACGACTTCTTCAATATTCACCGAGC
CTTCGCTGCACTGCTTTAGCTGCAGCTTCACCAGAACTGCTGCATAGGCTGATACCAGAGCATGTCCGC
CGGCAAGTTGGTCTCAGTTTCCCCCATCCTGCTGGCACGTAA

> *VviBIN2.3*_VIT_214s0060g00600
ATGGCTTCAGTGGGCATAGCACCTACCTCTGGTTTGAGAGAACCTAGTGGCCACAATGCCGGTGTTGA
TAGGTTGCCAGAAGAGATGAATGACATGAAAATTAGGGATGACAAAGAGATGGAAGCAACAGTTGTT
GACGGTAATGGAACAGAGACAGGTCATATAATTGTGACGACTATTGGTGGTAGAAATGGCCAACCAA
AGCAGACAATAAGTTACATGGCTGAGCGCGTTGTTGGGCATGGATCATTTGGAGTTGTTTTTCAAGCA
AAATGCTTAGAGACCGGTGAAACTGTGGCTATAAAGAAGGTTCTTCAAGACAAGAGATATAAGAACC
GTGAACTTCAAACTATGCGTCTTCTTGACCACCCTAATGTTGTCTCCTTGAAGCACTGTTTCTTTTCAAC
AACCGAAAAGGATGAACTTTATCTTAATCTTGTACTTGAGTATGTTCCTGAAACTGTTCATCGGGTTAT
TAAACACTACAATAAGTTGAACCAAAGGATGCCACTGATATATGTGAAGCTCTATACATACCAGATTT
TCAGGGCATTGTCTTACATTCATCGCAGTATTGGAGTTTGTCATAGGGACATCAAACCTCAAAATCTTT
TGGTGAATCCACATACCCACCAGCTTAAGCTATGTGACTTTGGAAGTGCAAAAGTCTTGGTAAAAGGG
GAACCCAATATATCTTATATCTGCTCTAGATATTACAGGGCTCCTGAGCTTATATTTGGAGCAACTGAG
TACACTACAGCTATTGATATCTGGTCTGCAGGTTGCGTTCTGGCTGAGTTAATGCTTGGACAGCCTCTG
TTTCCTGGAGAGAGTGGAGTCGATCAGCTTGTTGAGATTATCAAGGTTTTGGGCACTCCAACAAGGGA
GGAAATTAAATGCATGAACCCCAACTATACAGAGTTCAAATTCCCCCAAATTAAAGCTCACCCATGGC
ACAAGATATTCCATAAACGCATGCCTCCAGAAGCTGTTGATTTGGTTTCAAGACTACTGCAGTACTCTC
CTAATCTACGGTGCACAGCTATGGATGCCTTGACCCATTCCTTCTTCGATGAGCTACGTGACTCAAGCA
CCCGCCTGCCAAATGGCCGATTTCTTCCACCACTATTCAACTTTAAGCCTCACGAATTGAAGGGAGTAC
CAGTTGAGATTTTGGTGAAATTGATCCCGGAACATGCAAGAAAGCAATGCCCCTTCCTTGGGCTATGA

> *VviBES1_* VIT_204s0023g01250
ATGGCTGGAGGCGGATCATCCGGAAGGTTGCCTACTTGGAAGGAGAGGGAGAACAACAAGAAGAGG
GAGAGGAGAAGAAGGGCCATAGCTGCTAAGATATACGCGGGGCTTAGAGCTCAGGGCAACTACAAGC
TTCCCAAGCACTGCGATAACAACGAGGTCTTGAAGGCTCTATGTTCTGAGGCTGGTTGGACCGTTGAA
GAAGATGGCACCACCTACCGCAAGGGATGCAAACCACCCCCAACGGAAATTGCAGGCGCTTCGGCCA
ACATCAGTGCATGTTCTTCCATGCAACCAAGCCCACAGTCCTCATCCTTCCCAAGTCCTGTACCCTCTT

ACCATGCCAGCCCATCGTCCTCCTCTTTCCCGAGCCCCACTCGGTTTGATGGAAACCCCTCTTCATACC
TCCTCCCTTTCCTCCGTAATTTAGCCTCGATTCCTACATCTCTCCCACCTCTTCGAATATCCAACAGTGC
TCCTGTAACCCCGCCTCTTTCTTCCCCTACTTCGAGAGGTTCAAAGCGGAAACCTGACTGGGAATCCTT
CTCAAATGGCTCCCTGAATTCCTTCCGCCACCCTCTATTTGCCGTATCAGCCCCCTCTAGTCCTACTCGC
CGCAACCATCTTACACCTGCCACAATACCGGAATGTGATGAATCTGATGCCTCTACTGTTGATTCTGGT
CGCTGGGTGAGTTTCCAGACAGTAGCACCCCAGGCAGCTCCATCTTCCCCTACATTTAATCTTGTAAAA
CCAGTGGCTATGGAGTGTTCTATCCCCAATGCAGTTGATGAGCATGGAGGTCTGGGCTGGGGTGCAGC
AGCAGAGAGGGGCCGGCCAGAGTTTGAGTTTGAGAGTGGCAGAGTGAAGGCGTGGGAGGGTGAGAG
AATTCATGAGGTAGGAGTGGATGAACTGGAGCTTACACTTGGGAGTGGGAAGACTCGTGGTTAA

> *VviBZR1*_ VIT_218s0001g12020
ATGACGTCAGAGAGGACGCCGACGAGGAGGAAGGCGTCGTGGAAGGAGAGGGAGAACAACATGAGG
AGAGAGAGGAGGAGGAGAGCCATAGCGGCAAAGATATATGCGGGCCTCAGGGCTCAGGGCAACTATC
GTCTTCCAAAACACTGCGATAACAACGAGGTCCTCAAGGCTCTCTGCTCCGAAGCTGGTTGGACCGTT
GAAGATGACGGCACCACCTATCGCAAGGGATGCAAGCCTCCCCCCTCAACTGAGATTGCAGGAACTTC
CACAAACAACACTCCCTGCTCTTCCCAGAAACCAAGCCCACCATCTTCCTCCTTTCCAAGCGCATTCGC
TTCCTACCAACCCAGTCCCTCATCCTCAAACCTGTCTTTCATGGATGCCAATGCCTCTCTCAATCTCCTT
CCATTTCTCTACAAGTCTATCCCTTCATCTCTGCCTCCTCTCCGAATATCAAACAGTGCTCCTGTAACAC
CACCTATTTCGTCCCCAACCTCCAGAGTTCCCATGCCTAAACCCAACTGGGAGTCCCTTGCCAAAGAAT
CCATGGCCTCTATCCATCACCATTACCCCATCTTTGCTGCTTCTGCCCCAGCAAGCCCTTCTCGCTGTCA
GTATATTGCTCCTGCCACTATACCTGAATATGAGGAGTCTGACACCTCAACTGTTGAGTCAGGCCAGTG
GGTGAGTTTTCAGACGTTTGCACGCCATCTTGCTCCATTGCCCCCAACCTTCAATCTTATGAAACCTGT
GGCTCAGAAAATTTCACCAGATGAAGCAACCAAAGAGAAGGGGATAACTCCCGAGTTGGAAATTGGA
AGTGCACAGGTGAAGCCCTGGGAAGGGGAGAGGATTCATGAGATAGGTTTGGATGATCTGGAGCTTA
CACTAGGAAGTGGAAAGAGTAGGAGTAAAGGCTAA
